# Protective effects of small RNAs encapsulated in *Artemisia Capillaris*-derived exosomes against non-alcoholic fatty liver disease

**DOI:** 10.3389/fphar.2024.1476820

**Published:** 2025-01-06

**Authors:** Min Xu, Longjun Ma, Hongwei Liang, Wei Tang, Shouyong Gu

**Affiliations:** ^1^ Geriatric Hospital of Nanjing Medical University, The Fourth Clinical Medical College of Nanjing Medical University, Nanjing, China; ^2^ Department of Epidemiology, School of Public Health, Nanjing Medical University, Nanjing, China; ^3^ Department of Emergency, Nanjing Drum Tower Hospital, School of Life Science and Technology, China Pharmaceutical University, Nanjing, China; ^4^ Department of Endocrinology, Geriatric Hospital of Nanjing Medical University, Nanjing, China; ^5^ Institute of Geriatric Medicine, Jiangsu Province Geriatric Hospital, Nanjing, China

**Keywords:** *Artemisia capillaris*, exosomes, nonalcoholic fatty liver disease, lipid metabolism, cross species regulation

## Abstract

**Introduction:**

*Artemisia capillaris*, a traditional medicinal plant, is renowned for its therapeutic properties, including the promotion of anti-inflammatory and bile secretion. Notably, it has demonstrated efficacy in the treatment of jaundice. This study aimed to evaluate the potential of *Artemisia capillaris*-derived exosomes (ACDEs) as a novel therapeutic approach in non-alcoholic fatty liver disease (NAFLD).

**Methods:**

The physicochemical properties of ACDEs were isolated and characterized using differential centrifugation, and the therapeutic efficacy was evaluated in an *in vivo* methionine-choline-deficient (MCD) diet induced NAFLD mouse model. *In vitro*, mouse hepatocytes were treated with palmitic acid (PA) to simulate a high fat environment. Intracellular triglycerides (TG) and total cholesterol (TC) levels were quantified, and Oil Red O staining was assessed. Additionally, the expression levels of proteins and RNAs associated with lipogenesis and inflammation were analyzed.

**Results:**

The NAFLD mouse model exhibited notable liver damage, including lipid deposition and inflammatory responses. However, treatment with ACDEs exhibited broad pharmacological activities, effectively reversing hepatic lipid accumulation and inflammatory damage. *In vitro* experiments revealed that ACDEs were internalized by AML12 cells via macropinocytosis and caveolin-mediated endocytosis. This treatment ameliorated dysregulated lipid metabolism and inhibited inflammatory responses. High throughput sequencing further identified a distinct small RNA profile in ACDEs, indicating the potential involvement in interspecies physiological regulation.

**Discussion:**

In conclusion, this study provides evidence for the therapeutic potential of ACDEs in NAFLD and offers a novel perspective for the development of Artemisia capillaris-based therapies for NAFLD, related metabolic disorders, and hepatitis.

## Graphical Abstract

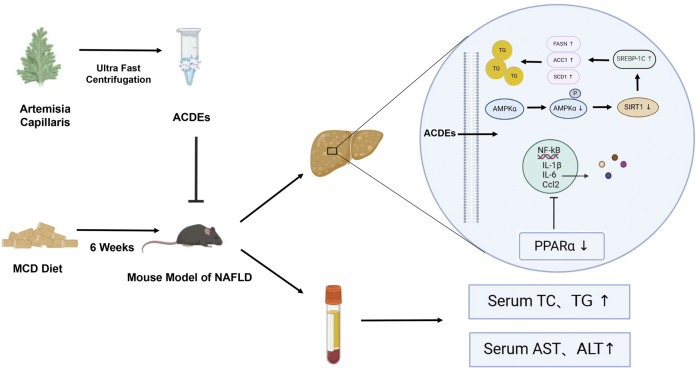

## 1 Introduction

Non-alcoholic fatty liver disease (NAFLD), a pervasive global health problem, is characterized by hepatic steatosis, inflammation, and oxidative stress, often progressing to nonalcoholic steatohepatitis (NASH), liver fibrosis, and hepatocellular carcinoma (HCC). It is associated with comorbidities like type 2 diabetes mellitus (T2DM) and cardiovascular diseases, posing a significant threat to human health (Zhou et al., 2021). Despite its prevalence, there are currently no targeted pharmaceutical interventions for NAFLD.

*Artemisia capillaris*, a member of the Asteraceae family, exhibits multiple physiological activities such as hepatoprotective, cholagogue, anti-inflammatory, and antioxidant effects (Liu et al., 2022), cementing its status as a staple in traditional Chinese medicine for the treatment of liver and gallbladder diseases (Xiong et al., 2021). Studies demonstrate its efficacy in ameliorating NAFLD and obesity in mice fed a high fat diet (Cai et al., 2019). This herb activates the PI3K/AKT and AMPK signaling pathways, leading to reduced expression of SREBP-1c and decreased hepatic fat synthesis and accumulation (Liang et al., 2022). Its bioactive constituents, such as artemisinin and chlorogenic acid, exhibit potent antioxidant and anti-inflammatory properties that are critical for alleviating oxidative stress and inflammation in liver diseases (Hsueh et al., 2021).

Plant-derived exosomes, nano-sized vesicles secreted by plant cells, are rich in a variety of biomolecules, including nucleic acids, proteins, lipids, and various specialized small molecules (Rome, 2019). These vesicles serve critical roles in intercellular communication, information exchange, and the maintenance of organismal homeostasis, as well as in disease therapy (Hu et al., 2023). In 2007, An et al. (2007) illuminated the secretion of extracellular vesicles by barley leaf cells, highlighting their structural and functional similarities to mammalian exosomes and their bioactive cargo. The unique vesicular structure of plant-derived exosomes provides a natural and environmentally friendly delivery platform, presenting significant potential in the targeted delivery of therapeutic agents (Yang et al., 2023).

In 2014, Mu et al. first investigated the exosome like nanoparticles (ELNs) derived from grapes, grapefruits, ginger, and carrots could be absorbed by mouse intestinal macrophages and intestinal stem cells (Mu et al., 2014). In 2022, a study indicated that exosomes derived from mesenchymal stem cells could effectively reduce hepatic steatosis in NAFLD (Kang et al., 2022). Additionally, Wang et al. (2022) also explored the mechanisms of exosomes in various liver diseases, including NAFLD, emphasizing their importance in disease progression and treatment (Guo et al., 2022).

Although *Artemisia capillaris* has been widely used in traditional Chinese medicine, the therapeutic activity of *Artemisia capillaris*-derived exosomes (ACDEs) remains unexplored, the role of ACDEs has not been documented in NAFLD. In this study, we first employed a differential centrifugation method to isolate and characterize ACDEs. Subsequently, we established a NAFLD model to investigate the role of ACDEs in this disease. Finally, we constructed and compared small RNA libraries between ACDEs and *Arabidopsis thaliana*. By performing target gene prediction and functional enrichment analysis on differentially expressed miRNAs in ACDEs compared to those in *A. thaliana*, we aimed to investigate the role and potential mechanism of ACDEs in NAFLD. Our findings promise to deepen the understanding of ACDEs from both a molecular and physiological perspective, offering substantial potential for precise drug delivery in the treatment of NAFLD.

## 2 Materials and methods

### 2.1 Isolation and extraction of exosomes from *artemisia capillaris*

*Artemisia capillaris* was obtained from a local pharmacy. The plant material was immersed in double-distilled water (ddH2O) at a ratio of 10:1 (water: material) for 1 h, followed by juicing and filtration to collect the filtrate. Exosomes were isolated using differential centrifugation through the following steps: 1,000 × g for 10 min, 3,000 × g for 20 min, and 10,000 × g for 40 min. The supernatant was further centrifuged at 120,000 × g for 70 min to pellet the exosomes. The supernatant was discarded, and the pellet was resuspended in phosphate-buffered saline (PBS). The resuspended pellet was sonicated to ensure uniform dispersion, forming a mixed solution, which was then filtered through a 0.22 µm filter to remove bacteria, thereby obtaining ACDEs. The protein concentration of ACDEs was determined using a bicinchoninic acid (BCA) assay kit (Beyotime). A standard curve was generated based on the absorbance values of different concentrations of bovine serum albumin (BSA) standard at 562 nm, and the protein concentration of ACDEs was calculated using the standard curve.

### 2.2 Animals and diets

Seven-week-old specific pathogen free (SPF) male C57BL/6J mice were procured from the Model Animal Research Center of Nanjing University Medical School (Jiangsu, China). Ethical approval for all animal procedures was obtained from the Nanjing Medical University Institutional Animal Care and Use Committee, and the study was conducted in strict accordance with the guidelines set forth by the Chinese Animal Welfare Committee. The mice were kept under SPF-grade conditions (25°C, 12-h light/dark cycle, 50% humidity) with unrestricted access to food and water. Following 1 week of acclimatization with a normal chow diet, the mice were randomized into three groups: 1), Normal Chow Diet group: Mice were fed with sterile, nutritionally balanced chow irradiated with 60 Co γ-rays for 6 weeks. 2), ACDEs Treatment group: Mice were fed with a methionine-choline-deficient (MCD) diet for 6 weeks and orally administered ACDEs (dissolved in saline at a dose of 7.8 g/kg body weight) every other day for 6 weeks. 3), MCD Diet (NAFLD) group: Mice were fed an MCD diet for 6 weeks and orally given saline every other day for 6 weeks. Body weight and fasting blood glucose were measured weekly for all groups of mice. At the end of the 6th week, blood was collected from the orbital sinus of each mouse, and serum was separated by centrifugation at 5000 g for 5 min. Additionally, liver tissues were harvested from each mouse and subsequently weighed.

### 2.3 ACDEs labelling

ACDEs were isolated via differential centrifugation. A 100 µg solution of ACDEs was combined with an appropriate volume of DIL red fluorescent dye to achieve a final concentration of 2 µM. The mixture was vigorously vortexed and then incubated at 37°C in the dark for 30 min. Excess dye was removed by ultracentrifugation at 120,000 × g for 70 min. The supernatant was discarded, and the exosome pellet was resuspended in PBS. The suspension was then sterilized by filtering through a 0.22 µm filter.

### 2.4 Biodistribution of ACDEs

DIL red fluorescent dye-labeled ACDEs were pre-prepared and dissolved in 200 µL of PBS solution. Four mice were randomly selected, and subjected to a 12-h fast. Each mouse was then orally administered 100 µg of DIL-labeled ACDEs. The mice were subsequently anesthetized at 0, 6, 12, and 24 h post-administration, respectively, and observed and photographed using the IVIS Spectrum system (PerkinElmer, USA). Post-imaging, the mice were euthanized with carbon dioxide, and the small intestines, livers, mesenteric lymph nodes, spleens, lungs, and kidneys were dissected for *ex vivo* imaging.

### 2.5 Cell culture and treatment

AML12 cells were cultured in a humidified incubator at 37°C and 5% CO_2_ with DMEM/F12 supplemented with Insulin-Transferrin-Selenium (1%), penicillin/streptomycin (1%), fetal bovine serum (10%), and dexamethasone (40 ng/mL). The cells were obtained from Suzhou Haixing Biological Technology Co., Ltd. The NAFLD cell model was established by exposing AML12 cells to 400 μM PA for 24 h (Peck et al., 2010). Subsequently, the corresponding reagents were added to the PA-induced NAFLD cell model culture medium (the ACDEs treatment group received 200 μg/mL ACDEs, while the control group received an equal volume of PBS solution). The cells were then cultured for an additional 24 h.

### 2.6 Measurement of AST, ALT, TC, and TG

The activity levels of alanine aminotransferase (ALT) and aspartate aminotransferase (AST) in serum were measured using an Applygen test kit (Applygen, Beijing, China). The activity levels of total cholesterol (TC) and triglycerides (TG) in cells, liver, and serum were measured similarly.

### 2.7 H&E staining and immunohistochemistry

The liver sections were initially washed with ultrapure water to eliminate the embedding agent, stained with hematoxylin for 4 min, differentiated with hydrochloric acid ethanol for 3 s, counterstained with ammonia for 1 min, and stained with eosin for 1 min. Subsequently, the sections were dehydrated, cleared, and sealed. Frozen sections were thawed at room temperature for 2 h, treated twice with xylene, and dehydrated in ethanol. After antigen retrieval, an endogenous peroxidase blocker was added and incubated for 10 min at room temperature. The primary antibody was applied overnight, followed by incubation with an enzyme-labeled secondary antibody and diaminobenzidine (DAB) for color development, lasting for 5 min. Finally, the sections were counterstained with hematoxylin, dehydrated, cleared, and sealed.

### 2.8 Oil red O staining

The cells were initially rinsed with PBS, subsequently fixed, and stained with Oil red O before observation under a fluorescence microscope. For frozen sections of liver tissue, the samples were covered with a staining washing solution after rewarming, followed by staining with Oil red O staining solution. Ultimately, these sections were observed and photographed directly under a fluorescence microscope (Axio Observer, ZEISS vision care, Germany).

### 2.9 Masson staining

Liver tissue was fixed in Bouin’s solution, rinsed in PBS overnight, and dehydrated for embedding. The sections were then deparaffinized to water, and stained with Weigert’s iron hematoxylin for 10 min. Following a rinse with PBS, the sections were differentiated with 1% hydrochloride ethanol, and washed with PBS for a few minutes. Subsequently, the sections were blued with Masson’s bluing solution, rinsed in PBS for 1 min, and stained with acid fuchsin for 10 min, followed by a brief rinse in PBS. The sections were then treated with phosphomolybdic acid for about 5 min, stained with aniline blue for 5 min, and then washed with 1% acetic acid until no blue color remained. Finally, sections were rapidly dehydrated with 95% ethanol and absolute ethanol (three times, 10 s each), cleared in xylene (three times, 10 min each), and mounted with neutral balsam.

### 2.10 RNA extraction and RT-qPCR

Total RNA was extracted from tissues and cells using Trizol reagent (Vazyme, Nanjing, China) according to the manufacturer’s instructions. The concentration and purity of total RNA were assessed using a Nanodrop spectrophotometer. Subsequently, 1 µg of total RNA was reverse transcribed into cDNA using the Reverse Transcription Kit (Vazyme, Nanjing, China) with HiScript II qRT SuperMix II and random primers. Real-time quantitative PCR was performed using the Taq Pro Universal SYBR qPCR Master Mix (Vazyme, Nanjing, China) with the following cycling parameters: initial denaturation at 95°C for 30 s, followed by 40 cycles of denaturation at 95°C for 10 s and annealing/extension at 60°C for 30 s. Relative mRNA expression levels were calculated using the 2^-ΔΔCt method, with Actin serving as the internal reference gene. The fold change in mRNA expression between treatment and control groups was determined. All primers were purchased from Tsingke, and details of the primers used for analysis are provided in Supplementary Table S1.

### 2.11 Western blotting

Total protein was extracted from cells or tissues using RIPA lysis buffer containing protease and phosphatase inhibitors and quantified using a BCA protein assay kit (Beyotime, Shanghai, China). Equal amounts of protein (30 μg) were separated by SDS-PAGE gels and transferred onto PVDF membranes. The membranes were blocked with 5% skimmed milk at room temperature for 1 h. Subsequently, the membranes were incubated with specific primary antibodies at 4°C overnight. primary antibodies used included the following: FASN (1:10,000, Proteintech, China), ACC1 (1:3,000, Proteintech, China), GAPDH (1:50,000, Proteintech, China), SCD1 (1:3,000, Proteintech, China), CD36 (1:500, Wanlei, China), SREBP-1c (1:1,000, Wanlei, China), AMPKα (1:1,000, Wanlei, China), P-AMPK (1:500, Wanlei, China), PPARα (1:500, MCE, China), IKKβ (1:500, Affinity Biosciences, China), P-IKKβ (1:500, Affinity Biosciences, China), P65 (1:500, Affinity Biosciences, China), and P-P65 (1:500, Affinity Biosciences, China). After primary antibody incubation, the membranes were washed and incubated with HRP-conjugated secondary antibodies at room temperature for 1 h. Protein signals were detected using an enhanced chemiluminescence (Beyotime, Shanghai, China) substrate and visualized with an imaging system (Tanon, Shanghai, China).

### 2.12 Small RNAs sequencing of ACDEs

Total RNA was extracted using Trizol, and its concentration was determined with a NanoDrop spectrophotometer. RNA integrity was assessed using a Bioanalyzer 2100. The extracted RNA was treated with a 3′adaptor at 70°C for 2 min and purified by ligation with T4 RNA Ligase two overnight at 16°C. Subsequently, the RNA was incubated with RTP reagent at 37°C for 30 min to neutralize and remove single-stranded DNA 3′ adapters. The ligation product was then subjected to further ligation with RNA 5′ adapters using T4 RNA ligase 1 at 37°C for 1 h, followed by purification. Reverse transcription was performed using SuperScript II (Thermo, 18064014, USA) at 50°C for 1 h, with an additional incubation at 80°C for 10 min. Second-strand synthesis and amplification were carried out using Phusion High-Fidelity DNA polymerase. The PCR cycling conditions included an initial denaturation at 98°C for 30 s, followed by 10–16 cycles of denaturation at 98°C for 10 s, annealing at 60°C for 30 s, and extension at 72°C for 15 s, with a final extension at 72°C for 5 min. The miRNA library was purified and enriched by polyacrylamide gel electrophoresis (PAGE), and single-end 50-bp sequencing (SE50) was performed using the Illumina HiSeq 2500 platform.

### 2.13 Statistical analysis

Statistical analyses were conducted utilizing GraphPad Prism 8 software. Data were presented as mean ± standard deviation (mean ± SD). Group Comparisons were analyzed using analysis of variance (ANOVA). Each experiment was replicated three times or more. *p* < 0.05 was considered statistically significant.

## 3 Results

### 3.1 Isolation and characterization of ACDEs

**FIGURE 1:**
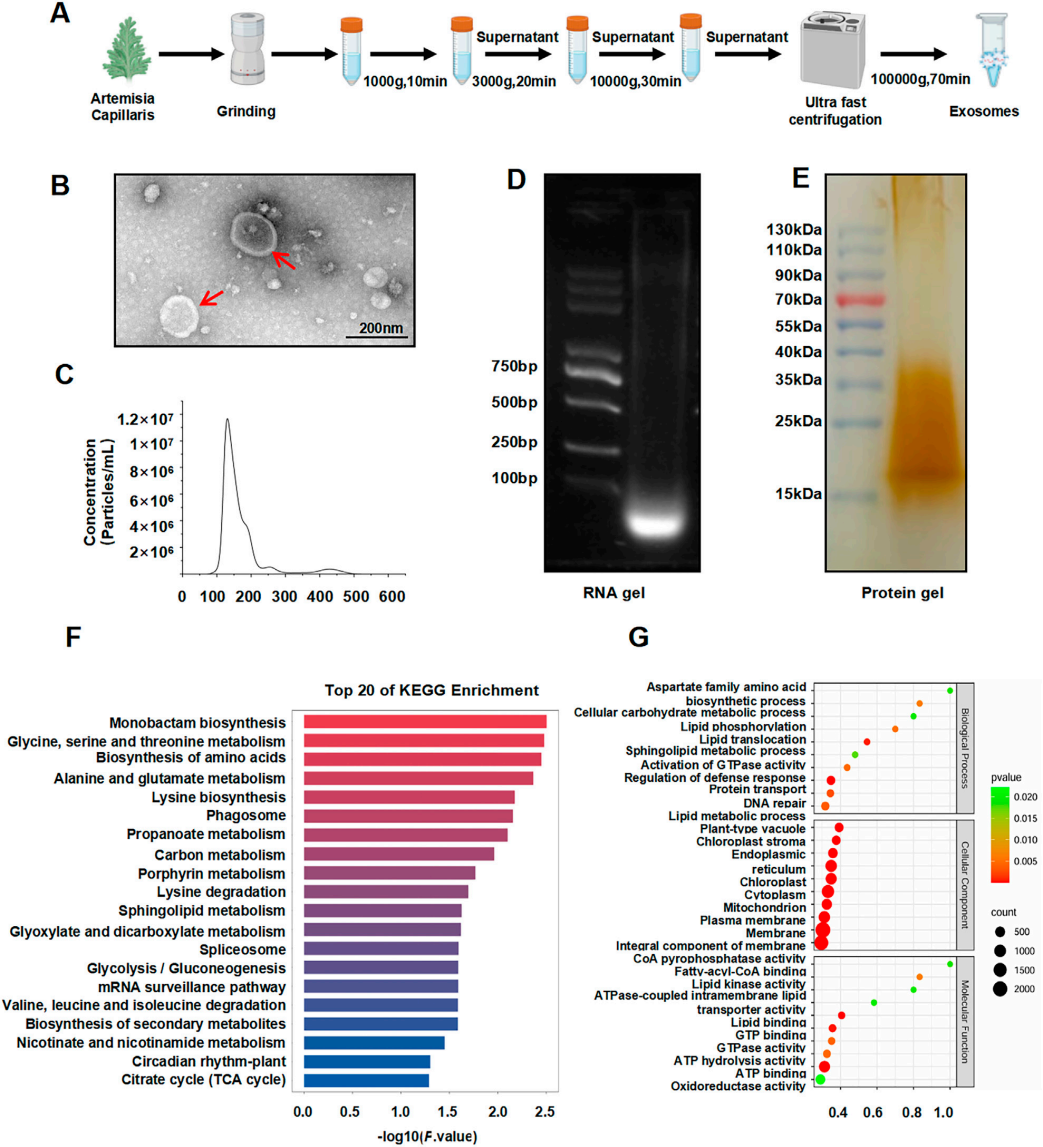
Isolation and characterization of ACDEs. **(A)** Schematic diagram illustrating the isolation of ACDEs. **(B)** Morphology of ACDEs analyzed by TEM. Scale bar represents 200 nm. **(C)** Size distribution analysis of ACDEs by NTA. **(D)** RNA extraction from ACDEs using a total RNA extraction kit, followed by 2% agarose gel electrophoresis and imaging using a gel imaging system. **(E)** Protein extraction from ACDEs, followed by 15% SDS-PAGE protein electrophoresis of 20 μg protein and silver staining for imaging. **(F)** GO analysis of target genes corresponding to differentially expressed miRNAs between ACDEs and Arabidopsis. **(G)** KEGG analysis of target genes corresponding to differentially expressed miRNAs between ACDEs and Arabidopsis.

ACDEs were successfully isolated via differential centrifugation (Figure 1A). Transmission electron microscopy (TEM) revealed a homogenous morphology with irregular circular shapes, central indentations, and bilayer membranes, resembling classical exosomal structures (Théry et al., 2018) (Figure 1B). Nanoparticle tracking analysis (NTA) demonstrated a size range of 134.9–317.1 nm, with an average diameter of 201.1 nm (Figure 1C). Agarose gel electrophoresis confirmed the presence of small RNAs (Figure 1D), while protein electrophoresis revealed the protein cargo (Figure 1E). These findings substantiate the exosomal nature of ACDEs, which are enriched with RNAs and proteins (Chen et al., 2023).

To characterize the miRNA profile of ACDEs, we compared the data with miRBase using *A. thaliana* as a reference and identified 293 miRNAs, including 196 downregulated and one upregulated miRNA, gma-miR5368. Target prediction using miRanda revealed that these differentially expressed miRNAs are involved in various cellular and metabolic processes, in particular lipid transport and phosphorylation (Figure 1F). KEGG enrichment analysis highlighted 20 pathways associated with these targets, including amino acid metabolism, lipid metabolism, and secondary metabolic pathways (Figure 1G). These findings suggest that ACDE miRNAs may regulate key metabolic processes, particularly those related to amino acid and lipid metabolism.

### 3.2 Effects of ACDEs on lipid metabolism and inflammatory factors in A NAFLD Cell model

**FIGURE 2:**
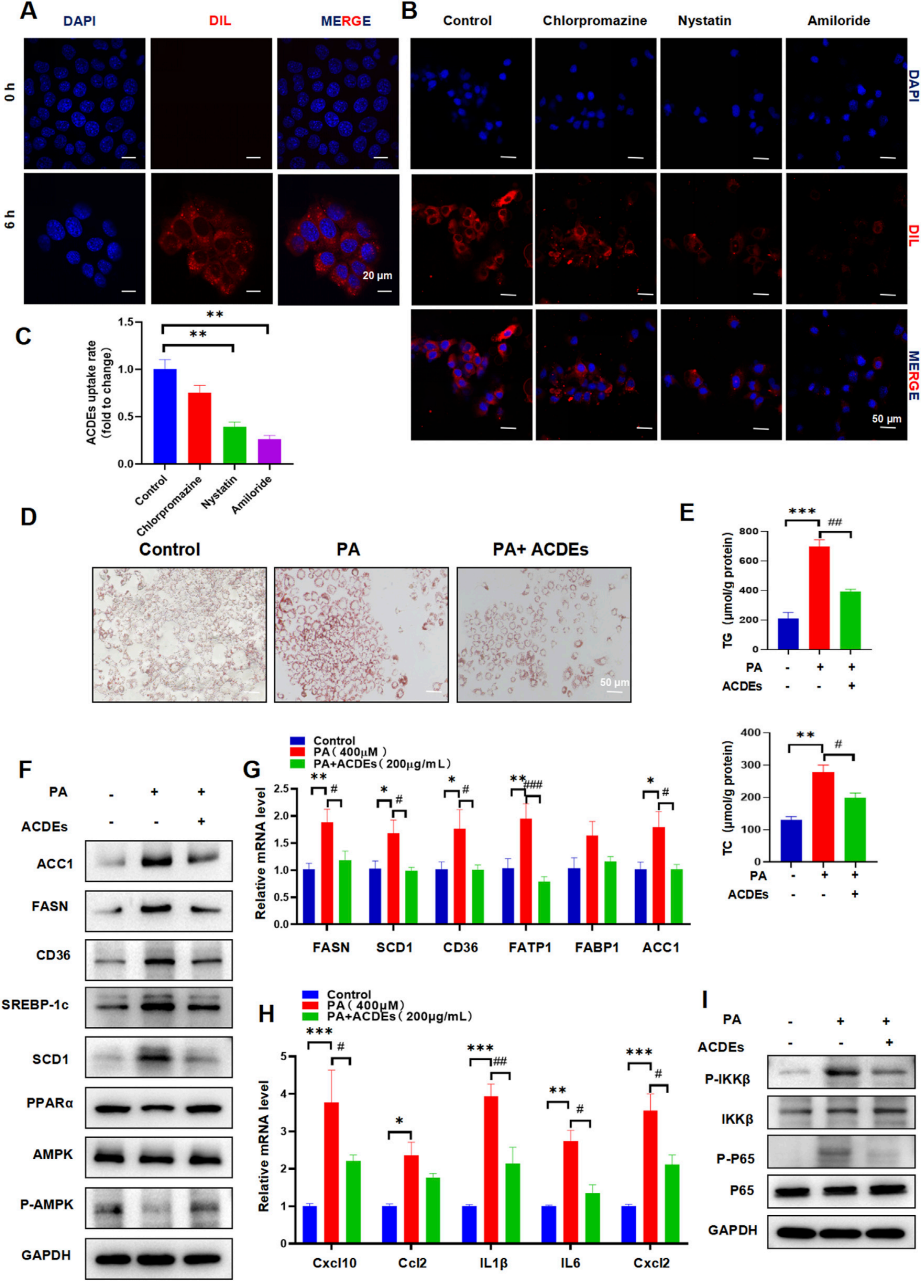
Induction of NAFLD Cell Model in AML-12 Cells by PA 400 μmol, Co-culture with ACDEs Improves Lipid Deposition and Alleviates Hepatic Dysfunction. **(A)** Uptake of ACDEs by NAFLD model cells. NAFLD cells were treated with DiI-labeled ACDEs for 0 and 6 h. Nuclei were stained with DAPI (blue). Images were captured using confocal microscopy. Scale bar represents 10 μm. **(B)** AML12 cells were pre-treated with nystatin, chlorpromazine, and amiloride for 1 h before being incubated with DIL-labeled ACDEs for 6 h. Cell nuclei were stained with DAPI(blue), while ACDEs were marked with DIL (red). **(C)** percentage of ACDE uptake compared to the control **(D)** Oil Red O staining after co-culture of ACDEs with NAFLD cells. **(E)** Levels of cellular TG and TC after co-culture of ACDEs with NAFLD cells. **(F)** Protein expression of FASN, ACC1, SCD1, SREBP-1c, CD36, PPARα and AMPK was examined by WB. **(G)** mRNA expression levels of FASN, SCD1, CD36, FATP1, FABP1, and ACC1 after co-culture of ACDEs with NAFLD cells. **(H)** The relative expression of Cxcl10, Ccl2,IL1β,IL6 and Cxcl2 was determined by qRT-PCR. **(I)** WB analysis was performed to evaluate the expression of NF-κB pathway-related proteins.*, # indicate significant differences between two groups, *** and ### represent *p* < 0.001, ** and ## represent *p* < 0.01, * and # represent *p* < 0.05. *: Control vs. PA, #: PA vs. PA + ACDEs. The blue column represents Control, red column represents PA; green column represents PA + ACDEs.

To evaluate the cellular uptake and effects of ACDEs on NAFLD we investigated their internalization by AML 12 cells. The ACDEs, labeled with DIL fluorescent dye (red), were co-incubated with AML 12 cells for 6 h. Confocal laser scanning microscopy revealed significant internalization of ACDEs, as evidenced by the prominent red fluorescence within AML 12 cells (Figure 2A). This indicates efficient uptake and localization of ACDEs in the cell cytoplasm. Endocytosis is a key mechanism by which target cells internalize exosomes (Gurung et al., 2021). To elucidate the specific pathways involved in ACDEs internalization in AML12 cells, we used three selective inhibitors to systematically assess the contribution of different endocytic routes. As shown in Figures 2B, C, pre-treatment with the classical macropinocytosis inhibitor amiloride (10 µM) significantly reduced the red fluorescence signal in AML12 cells. Similarly, nystatin (25 μg/mL), a caveolin-mediated endocytosis inhibitor, reduced ACDEs internalization by more than 60%. In contrast, chlorpromazine has a minimal impact on the uptake of ACDEs by AML12 cells. These results indicate that ACDEs are primarily internalized by AML12 cells through mechanisms involving macropinocytosis and caveolin-mediated endocytosis.

An NAFLD cell model was established by treating AML12 cells with 400 μM PA for 24 h (Peck et al., 2010). ACDEs (200 μg/mL) were then added to the treatment group, while the control group received PBS, and cells were cultured for another 24 h. Oil Red O staining confirmed extensive lipid deposition in PA-treated cells, while ACDEs significantly reduced the size and number of lipid droplets (Figure 2D). Consistently, ACDEs treatment substantially decreased PA-induced elevations in TG and TC levels (Figure 2E). Western blot analysis showed that PA stimulation increased the expression of FASN, ACC1, SCD1, SREBP-1c, and CD36 while reducing PPARα and P-AMPK/AMPK levels. ACDEs reversed these effects, decreasing lipogenesis-related proteins and increasing PPARα and P-AMPK/AMPK expression (Figure 2F). Gene expression analysis further revealed that PA upregulated FASN, SCD1, CD36, FATP1, FABP1, and ACC1 mRNA levels, whereas ACDEs significantly downregulated FASN, SCD1, CD36, FATP1, and ACC1, demonstrating their inhibitory effect on PA-induced lipid accumulation (Figure 2G).

Chronic inflammation is a key factor in the pathogenesis of NAFLD. During NAFLD progression, pro-inflammatory cytokines typically increase. We assessed the mRNA levels of Cxcl10, Ccl2, IL-1β, IL-6, and Cxcl2 in hepatocytes treated with palmitic acid (PA) and observed a significant upregulation of these inflammatory mediators following PA stimulation. However, treatment with ACDEs effectively suppressed the PA-induced expression of these cytokines (Figure 2H). Since the NF-κB pathway plays a crucial role in pro-inflammatory signaling, we further investigated its activation in liver inflammation. Our findings revealed that PA stimulation elevated the phosphorylation levels of IKKβ and p65, while the levels of their non-phosphorylated forms remained unchanged, indicating activation of the NF-κB pathway by PA. Importantly, ACDE treatment inhibited this NF-κB signaling cascade (Figure 2I).

### 3.3 ACDEs biodistribution in mice

**FIGURE 3:**
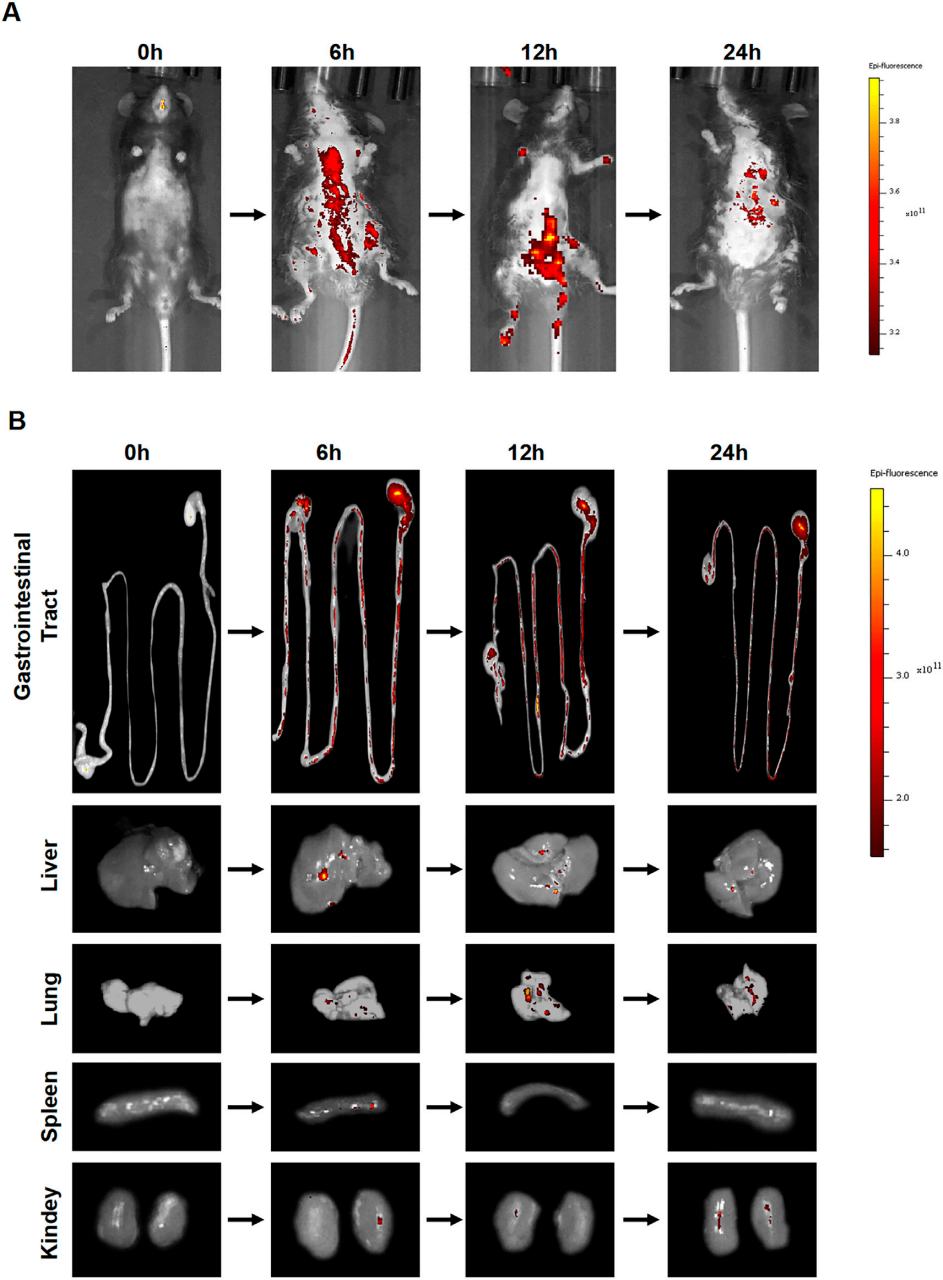
Biodistribution of ACDEs in Mice. **(A)** Each mouse was administered DIL-labeled ACDEs by gavage and observed using an *in vivo* imaging system at 0 h, 6 h, 12 h, and 24 h post-administration for photography. **(B)** The DiI fluorescence signal was primarily detected in the gastrointestinal tract and liver, peaking at 6 h. By 12 h, fluorescence signals were also observed in the mesenteric lymph nodes and lungs. By 24 h, there was a significant decline in the fluorescence signal *in vivo*.

To delineate the *in vivo* distribution of ACDEs, we randomly assigned four mice to a 12-h fasting period before administering DIL-labeled ACDEs via oral gavage. The biodistribution and absorption of ACDEs were subsequently evaluated using an *in vivo* imaging system. DIL, a lipophilic dye that markedly amplifies fluorescence intensity upon binding to ACDEs, is frequently employed *in vivo* imaging studies. In this experiment, mice received DIL-labeled ACDEs at 0, 6, 12, and 24 h post-administration, and were imaged at these time points. ACDEs were effectively absorbed by the mice, with peak fluorescence intensity observed at 6 h post-administration (Figure 3A). The DIL fluorescence signals were prominently localized in the gastrointestinal tract and liver, achieving their highest levels at 6 h. By 12 h, fluorescence signals extended to the mesenteric lymph nodes and lungs, with a notable decline in signal intensity by 24 h (Figure 3B).

### 3.4 Effects of ACDEs on lipid metabolism and liver function in A NAFLD mouse model

**FIGURE 4:**
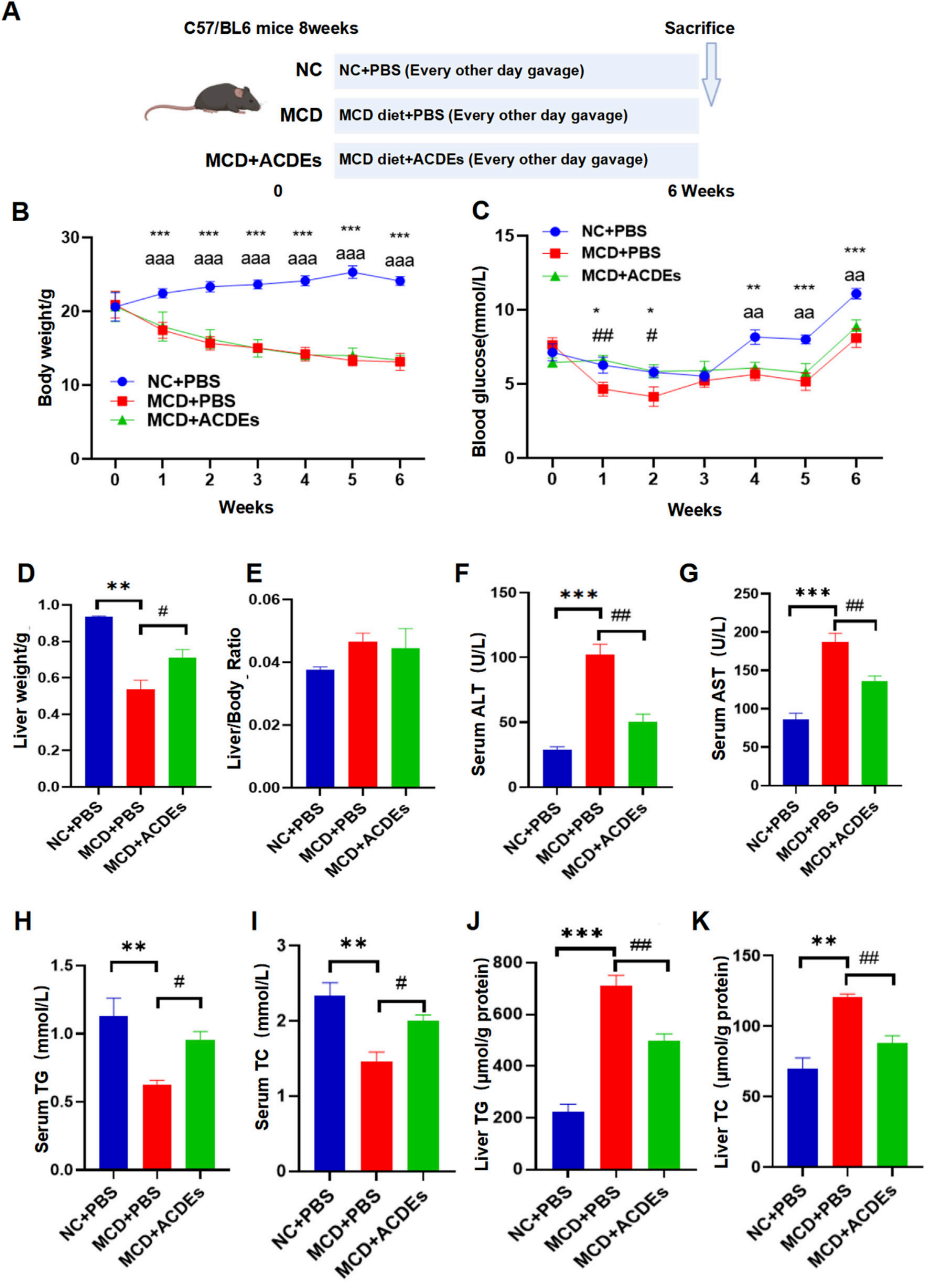
Effects of ACDEs on Lipid Metabolism and Liver Function in A NAFLD Mice. **(A)** Schematic diagram of NAFLD mouse modeling, **(B)** Monitoring of body weight in each group, **(C)** Monitoring of blood glucose in each group, **(D)** Liver weight of each group, **(E)** Liver weight ratio (liver weight to body weight) in each group. **(F)** Serum ALT levels in each group, **(G)** Serum AST levels in each group, **(H)** Serum TG levels in each group. **(I)** Serum TC levels in each group, **(J)** Liver TG levels in each group, **(K)** Liver TC levels in each group. *, #, a indicate significant differences between two groups, ***, ### and aaa represent *p* < 0.001, **, ## and aa represent *p* < 0.01, *, # and a represent *p* < 0.05. *: NC + PBS vs. MCD + PBS; #: MCD + PBS vs. MCD + ACDEs; **(A)** NC + PBS vs. MCD + ACDEs. The blue column represents Control group, red column represents PA; green column represents PA + ACDEs.

To evaluate the impact of ACDEs on lipid metabolism and liver function in NAFLD mice, three groups were established: control, NAFLD model, and ACDEs treatment. The control group was fed a standard diet for 6 weeks, while the NAFLD model group received a MCD diet. The ACDEs group received ACDEs (7.8 g/kg via oral gavage every other day) alongside the MCD diet (Figure 4A). After 6 weeks, the control group had a mean body weight of 24.12 g, significantly higher than the NAFLD and ACDEs groups, which weighed 13.16 g and 13.38 g, respectively (*p* < 0.001), with no significant difference between the latter two (Figure 4B). Fasting blood glucose levels in the NAFLD group were consistently lower than the control group, except for the first 2 weeks when ACDEs temporarily elevated glucose levels. From the fourth week, ACDEs lowered fasting glucose levels compared to the NAFLD group (Figure 4C). Liver weights in the NAFLD group were 0.4 g lower than the control group. ACDEs treatment increased liver weight by 0.176 g compared to untreated NAFLD mice, though no significant differences were observed in liver weight ratios across groups (Figures 4D, E). The NAFLD group exhibited elevated serum AST and ALT levels, indicating liver dysfunction. ACDEs treatment significantly reduced AST and ALT levels, suggesting hepatoprotective effects (Figures 4F, G). Lipid analysis showed the MCD diet decreased serum TG and TC levels but increased hepatic lipid concentrations. ACDEs normalized serum TG and TC levels and significantly reduced hepatic lipid accumulation after 6 weeks (Figures 4H–K).

**FIGURE 5:**
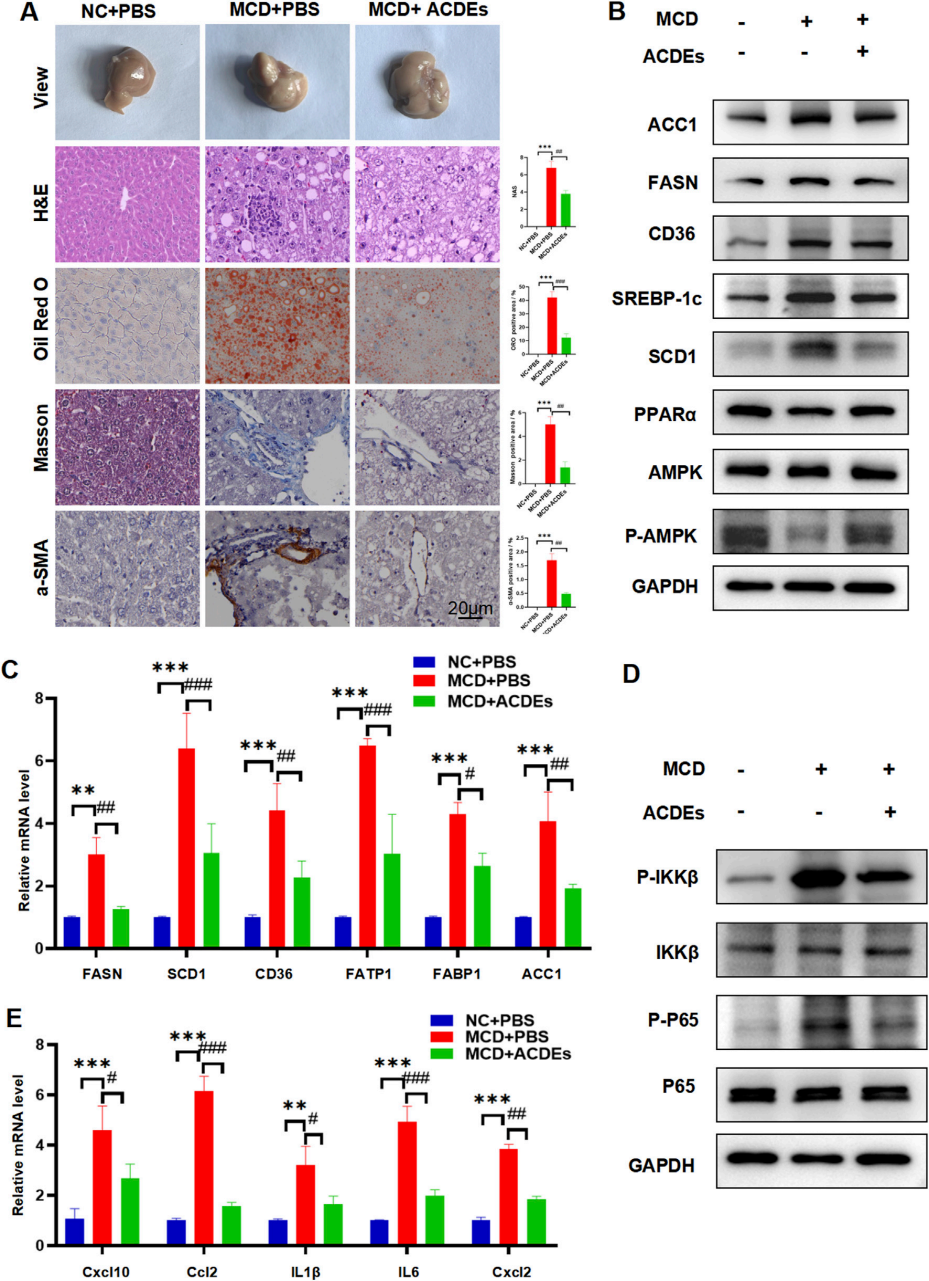
Effect of ACDEs on Hepatic Lipid Metabolism and Inflammation Levels in NAFLD Mice. **(A)** Histopathological observations of liver sections from each group of mice stained with HE, ORO, Masson’s trichrome, and α-SMA. **(B)** Protein expression of FASN, ACC1, SCD1, SREBP-1c, CD36, PPARα and AMPK was measured in the liver tissues of mice. **(C)** mRNA expression levels of FASN, SCD1, CD36, FATP1, FABP1, and ACC1 in liver tissues from each group of mice. **(D)** WB analysis was conducted to evaluate the expression of NF-κB pathway-related proteins. **(E)** mRNA expression levels of Cxcl10, Ccl2, IL-1β, IL-6, and Cxcl2 in liver tissues from each group of mice. *, # indicate significant differences between two groups, *** and ### represent *p* < 0.001, ** and ## represent *p* < 0.01, * and # represent *p* < 0.05. *: NC + PBS vs. MCD + PBS, #: MCD + PBS vs. MCD + ACDEs. The blue column represents Control group, red column represents PA; green column represents PA + ACDEs.

To observe the effects of ACDEs on liver morphology in the NAFLD mice, we compared liver tissues across the three groups. Livers from the NAFLD group showed blunted edges and substantial white fat deposition, whereas the control group livers appeared healthy. 6 weeks of ACDEs treatment significantly reduced lipid deposition in the liver (Figure 5A). H&E staining revealed numerous vacuoles in the NAFLD group, indicative of hepatic steatosis, while the ACDEs treated group showed a marked reduction in vacuoles, indicating improved hepatic steatosis (Figure 5A). Oil red O staining confirmed a significant reduction in lipid accumulation in the ACDEs treated group. To evaluate fibrosis, α-SMA and Masson staining showed substantial collagen deposition in the NAFLD group, but a marked reduction in collagen levels in the ACDEs group (Figure 5A).

In accordance with our *in vitro* findings, liver tissues from NAFLD mice on the MCD diet showed significantly increased protein levels of FASN, ACC1, SCD1, SREBP-1c, and CD36, and decreased levels of PPARα and P-AMPK/AMPK. The ACDEs treatment resulted in a notable reduction in FASN, ACC1, SCD1, SREBP-1c, and CD36 expression, with elevated PPARα and P-AMPK/AMPK levels (Figure 5B). To further investigate the mechanisms, we measured the mRNA levels of fatty acid synthesis and transport genes (FASN, SCD1, CD36, FATP-1, FABP-1, ACC1) in liver tissues using qRT-PCR. Mice on the MCD diet showed significantly upregulated expression of these genes, but after 6 weeks of ACDEs treatment, mRNA levels of FASN, SCD1, CD36, FATP-1, and ACC1 were significantly downregulated (Figure 5C).

ACDEs treatment decreased the phosphorylation of pathway components in the liver tissue of NAFLD mice, significantly inhibiting the excessive activation of the NF-κB pathway induced by MCD stimulation (Figure 5D). Our findings suggest that ACDEs can inhibit the NF-κB signaling cascade and reduce lipid accumulation in the liver tissue of NAFLD mice. We also assessed the expression levels of pro-inflammatory cytokines in the livers of mice using qRT-PCR (Figure 5E). Compared to the control group, the mRNA expression levels of C-X-C motif chemokine ligand 10 (CXCL10), C-C motif chemokine ligand 2 (CCL2), Interleukin-1 beta (IL-1β), Interleukin-6 (IL-6), and (CXCL2)were significantly upregulated in the livers of the NAFLD group. After 6 weeks of ACDEs treatment, the mRNA levels of CXCL10, CCL2, IL-1β, IL-6, and CXCL2 were reduced. These findings suggest that ACDEs could modulate the expression of pro-inflammatory cytokines in the livers of NAFLD mice induced by 6 weeks of the MCD diet. These results confirm the protective effects of ACDEs against MCD-induced lipid metabolic disorders and liver inflammation *in vivo*.

## 4 Discussion

Plant-derived exosomes are nano-sized vesicles secreted by multivesicular bodies, crucial for intercellular communication and immune regulation in plants, aiding resistance to pathogens (Rome, 2019). Compared to animal exosomes, plant exosomes contain a wider range of proteins and nucleic acids, with lower toxicity and immunogenicity, making them promising for biomedical applications (Kim et al., 2022). Various methodologies exist for the isolation of exosomes, each offering distinct advantages (Feng et al., 2023). Differential centrifugation, a widely used method for exosome isolation, effectively separates sample components by centrifugal speed, yielding exosome-rich precipitates (Coumans et al., 2017). This method preserved the structural integrity, size, and morphology of ACDEs, enriching them with biologically active RNA and proteins. Particle size analysis via TEM and NTA validated the efficacy of this extraction method for Artemisia capillaris exosomes, aligning with established plant exosome identification protocols in the literature (Guo et al., 2023).

This study investigated the absorption and distribution of ACDEs in mice following gavage. Red fluorescence, marked by DIL labeling, was detected in the abdominal cavity 6–24 h post-gavage, peaking at 6 h, suggesting optimal absorption. ACDEs were found in organs such as the small intestine, liver, and lungs, indicating systemic absorption through the gastrointestinal tract. These findings support the potential use of ACDEs in drug delivery and therapy. NAFLD was induced in mice by an MCD diet for 6 weeks, leading to significant lipid accumulation, liver fibrosis, impaired liver function, and elevated expression of inflammatory cytokines and lipid metabolism genes. ACDEs treatment reduced lipid deposition, improved liver function, and alleviated fibrosis, while inhibiting pro-inflammatory factors. ACDEs also modulated lipid metabolism proteins, promoting fatty acid oxidation and inhibiting lipogenesis. *In vitro*, ACDEs alleviated lipid deposition and liver dysfunction in hepatocytes. Overall, ACDEs from Artemisia capillaris effectively improve lipid metabolism, reduce inflammation, and combat fibrosis in NAFLD.

NAFLD models can be established using dietary, chemical, or genetic methods, each with unique advantages and limitations. Chemical models often present systemic toxicity risks and fail to fully replicate NAFLD pathology, while genetic models, though accurate, are costly and may not capture the complexity of human disease. We chose a dietary induction model, considering high-fat (HFD), high-fat high-cholesterol (HFHC), and methionine-choline-deficient (MCD) diets. While HFD models require long periods to induce fatty liver and fibrosis, and HFHC models can introduce confounding metabolic issues, the MCD diet rapidly induces hepatic steatosis and inflammation. This model is widely recognized for effectively replicating NAFLD, making it the preferred choice for our C57BL/6J mouse model. We recognize that using a single model and dietary regimen may limit the generalizability of our findings, and future studies will incorporate additional models, such as db/db and HFD, to enhance the applicability and clinical relevance of our results.

Small RNAs, typically 20–30 nucleotides long, play key roles in various biological processes (Zhou et al., 2022). miRNAs, a major subset found in exosomes, regulate signaling pathways by targeting mRNAs or inhibiting translation, drawing significant attention (Luo et al., 2024). Recent advancements in high-throughput sequencing and bioinformatics have revealed that plant miRNAs regulate human immune and metabolic functions (Zhang et al., 2019). Using small RNA sequencing, we identified 293 miRNAs in ACDEs, with gma-miR5368 being the most abundant compared to Arabidopsis miRNAs. gma-miR5368 has been shown to target enzyme genes involved in metabolic pathways and regulate secondary metabolites in soybean, crucial for antioxidant capacity and pest defense (Owusu Adjei et al., 2021). Differential targeting analysis indicated that these miRNAs are involved in key cellular processes and lipid metabolism. Although this study did not validate individual miRNAs, future research will focus on examining the role of gma-miR5368 in lipid metabolism regulation. Specifically, we plan to co-incubate ACDEs with the NAFLD Cell Model, measure gma-miR5368 expression, and perform inhibition or overexpression experiments to assess whether ACDEs can serve as stable delivery vehicles for miRNAs and explore the relationship between gma-miR5368 and lipid accumulation.

In this study, differential centrifugation was used to isolate ACDEs with optimal morphology and particle size. ACDEs demonstrated therapeutic effects in both *in vitro* and *in vivo* NAFLD models, reducing lipid accumulation and improving inflammation. However, these findings are preliminary, and further validation in additional models and experimental conditions is necessary. High-throughput sequencing revealed that differentially expressed miRNAs in ACDEs may regulate key biological processes, particularly amino acid and lipid metabolism. Although ACDEs show promise in treating NAFLD, further research is needed to clarify their mechanisms. Future studies should use proteomics and signaling pathway analysis to elucidate the exact mechanisms of ACDEs, with RNA-binding protein immunoprecipitation (RIP) assays used to validate miRNA-target interactions. Additionally, exploring ACDEs as a stable delivery vehicle for miRNAs in AML12 cells will be a focus of future research. These findings enhance our understanding of ACDEs in NAFLD and lay the foundation for further exploration of plant-derived exosomes.

## Data Availability

The original contributions presented in the study are included in the article/Supplementary Material, further inquiries can be directed to the corresponding authors.
