# Supplementary material for: Protective effects of small RNAs encapsulated in Artemisia Capillaris-derived exosomes against non-alcoholic fatty liver disease
Source: Front Pharmacol. 2025 Jan 6;15:1476820. doi: 10.3389/fphar.2024.1476820 (PMC11743690; doi:10.3389/fphar.2024.1476820)
Supplement: Supplementary file 1 [file Table1.docx]

Table 1. Primers used for qRT-PCR

| **Gene name** | **Forward primer sequence** | **Reverse primer sequence** |
| --- | --- | --- |
| Fasn | AGGTGGTGATAGCCGGTATGT | TGGGTAATCCATAGAGCCCAG |
| Scd1 | TTCTTGCGATACACTCTGGTGC | CGGGATTGAATGTTCTTGTCGT |
| Cd36 | ATGGGCTGTGATCGGAACTG | GTCTTCCCAATAAGCATGTCTCC |
| Fatp1 | TCTGTTCTGATTCGTGTTCGG | CAGCATATACCACTACTGGCG |
| Fabp1 | GTCAGAAATCGTGCATGAAGGG | GAACTCATTGCGGACCACTTT |
| Acc1 | GGCCAGTGCTATGCTGAGAT | AGGGTCAAGTGCTGCTCCA |
| Cxcl10 | CCAAGTGCTGCCGTCATTTTC | TCCCTATGGCCCTCATTCTCA |
| IL1β | GAAATGCCACCTTTTGACAGTG | TGGATGCTCTCATCAGGACAG |
| IL6 | CTGCAAGAGACTTCCATCCAG | AGTGGTATAGACAGGTCTGTTGG |
| Ccl2 | TAAAAACCTGGATCGGAACCAAA | GCATTAGCTTCAGATTTACGGGT |
| Cxcl2 | CCAACCACCAGGCTACAGG | GCGTCACACTCAAGCTCTG |
